# Quantifying the spatial scales of animal clusters using density surfaces

**DOI:** 10.1098/rsif.2025.0274

**Published:** 2025-09-24

**Authors:** Max van Mulken, Jasper Eikelboom, Kevin Verbeek, Bettina Speckmann, Frank Van Langevelde

**Affiliations:** ^1^ Algorithms, Eindhoven University of Technology, Eindhoven, The Netherlands; ^2^ Wildlife Ecology and Conservation Group, Wageningen University and Research, Wageningen, The Netherlands; ^3^ Laboratory of Geo-information Science and Remote Sensing, Wageningen University and Research, Wageningen, The Netherlands

**Keywords:** animal groups, density, herds, kernel density estimation, spatial analysis, summary statistic

## Abstract

Animal clustering takes place at a variety of spatial scales. While methods to quantify clustering already exist, many of these methods are either scale independent, not parameter-free, or model proximity as a binary function, which makes them unsuitable for anisotropic systems and is not representative of the perception neighbourhood of animals. We describe a method to quantify the degree of clustering of point-location data at different spatial scales, which uses kernel density estimation to construct a density function from the underlying point-location data. We build upon this method to automatically detect cluster diameters using smoothing kernels that better represent the perception neighbourhood of animals. Finally, we test our methods on artificial datasets with varying clustering characteristics, as well as on a dataset of African bush elephants. Our method correctly assigns higher clustering values to spatial scales with high degrees of clustering and accurately outputs a set of spatial scales that correspond to cluster diameters. The accuracy of our method is insensitive to the chosen kernel function. Combined with the parameter-free nature of our method, this allows for easy detection of clustering scales in anisotropic and hierarchically clustered systems, such as animal groups.

## 1. Introduction

Animals are often neither randomly nor regularly distributed. Detecting patterns in animal distributions is an important goal in ecology, as these patterns allow us to make inferences about the underlying stimuli [1]. One ubiquitous pattern is that of clustering: animals, often conspecifics, that stay within close spatial proximity of each other for a period of time. Whether it be ungulates gathering in herds, fish in schools or insects in swarms, the wide range of species exhibiting this behaviour triggered a lot of research explaining these patterns [2].

One generally accepted benefit for prey animals is that gathering in groups reduces predation risk [3]. The reasons for this may be threefold. Firstly, in the event of a predator encounter, the proximity to conspecifics reduces the probability of being attacked by a predator simply due to the fact that the predator has a larger choice of prey, i.e. the dilution effect [4–6]. Secondly, being in the presence of a large number of prey animals means each individual gets to benefit from the collective vigilance of the group [5,7,8]. Whenever a predator draws near, each animal no longer has to rely on merely its own vigilance, but can be alerted by its group members, i.e. the many eyes effect. The benefits of collective vigilance extend beyond mere predation risk, allowing members of the group to forage more efficiently, for example [9]. Thirdly, large groups of prey animals are more capable of performing anti-predatory behaviour such as mobbing [10], allowing them to overwhelm and confuse the predator.

Aggregation can also be beneficial for predators; hunting in groups allows carnivores to hunt down large prey animals that they may not be able to hunt down by themselves [3,11]. In these situations, the potential gain in foraging efficiency outweighs the cost of having to share the gathered resources with other members of the group [3]. Another perceived benefit is that animals living in groups have a much easier time finding potential mates than those living solitary [3].

Clustering behaviour of a group of animals is thought to reflect underlying stimuli. For example, some prey animals may cluster more closely together upon detection of a threat [12,13]. This means that the detection and comparison of a group’s characteristics at different moments in time allows us to make inferences about group behaviour.

Detecting clustering in animals, however, is not a trivial task, as clustering can occur at different spatial scales. For example, when a large number of animals group together, several subgroups may appear within this large group (e.g. family groups). Our goal will be to develop a method that is able to detect this clustering at different spatial scales in a parameter-free manner. There are several methods available to detect and analyse clustering in spatial datasets. The output of existing methods can roughly be divided into two categories: *summary characteristics*, which attempt to summarize global patterns in the data using a single or few metric(s), or *descriptive characteristics*, which capture patterns in the dataset in a slightly more complex manner, for example, using data labels.

An example of a method that produces descriptive characteristics is *k-means clustering* [14] that attempts to, given an input parameter k, partition the data into k clusters such that there is a high degree of spatial auto-correlation within each cluster. This clustering approach starts by finding k data points to function as cluster centres and then assigns each other data point a label associated with one of these centres.

Another popular method of producing similar descriptive characteristics is *density-based spatial clustering of applications with noise* (DBSCAN) [15]. DBSCAN creates clusters of data points based on their spatial proximity to other data points, without requiring prior knowledge about the number of clusters in the data. Given two input parameters ϵ and m, DBSCAN considers a group of at least m data points as a cluster if the union of discs of radius ϵ centred around each data point forms a single connected component. Any data point that is not in a cluster is considered noise. DBSCAN mainly performs well for datasets in which clusters are spatially well separated.

These two clustering methods share a common downside: their performance is greatly dependent on the value of their input parameters. As such, prior knowledge of certain aspects of the data is required for these methods to work well. For k-means clustering, the number of clusters in the data must be known, whereas DBSCAN requires knowledge of both the minimum size of each cluster (m) and an estimation of the spatial scale of the clustering (ϵ). For DBSCAN, especially the latter is problematic. A very small value of ϵ will result in barely any clusters being found, whereas a very large value of ϵ will result in the entire dataset being identified as a single cluster. Furthermore, if a dataset has multiple clusters at different spatial scales and with different densities, DBSCAN is unable to detect all of them. As such, picking suitable values for these input parameters is non-trivial.

There is a large number of summary characteristics that can be used to characterize clustering in spatial datasets. One such example is the *Hopkins statistic* [16], which aims to measure clustering by testing the similarity of the data distribution to a uniformly distributed dataset. The measure of clustering in a dataset is then perceived to be inversely proportional to its similarity to a uniform distribution. This is achieved by measuring and taking the sum of the distance to the nearest neighbour of each data point. This summation is then compared with what its value would be in a uniformly distributed dataset. Because the Hopkins statistic merely measures the deviation from uniform, however, it does not necessarily indicate that a dataset is clustered. For example, the Hopkins statistic does not verify whether a dataset follows a multi-modal distribution or whether the perceived clusters in the data are well separated. Additionally, the Hopkins statistic requires a fixed domain, but is completely scale independent, which means it cannot meaningfully distinguish between clustering at different spatial scales.

A summary characteristic that does take scale into account is the popular *Ripley’s K function* [17]. Similarly to the Hopkins statistic, the K function characterizes clustering as the degree of deviation from a uniform distribution. Where the Hopkins statistics only looks at nearest neighbours, however, the K function considers which fraction of the dataset lies within the spatial neighbourhood of radius s around each point, for some input parameter s>0. Formally, the K function is defined as [17]


$$K(s)=\lambda^{-1}\sum_{i}\sum_{j\neq i}\frac{I(d_{i,j}<s)}{n},$$

where $d_{i,j}$ is the Euclidean distance between the ith and jth point in a spatial point set, λ indicates the expected event density and where I is the *indicator function* that is equal to 1 if $d_{i,j}<s$ and 0 otherwise. Often, the expected event density is estimated as $\lambda =\frac{n}{A}$, where n is the total number of points and A is the area of the domain. Intuitively, Ripley’s K function thus compares the expected density of a uniformly distributed dataset using the local density in the neighbourhood of each point. If the spatial dataset is approximately homogeneous, the value of K(s) is approximately $\pi s^{2}$[18]. As such, when $K(s)-\pi s^{2}>0$, the dataset is considered to be more clustered than uniformly distributed data. The derivative of Ripley’s *K* function is proportional to the pair correlation function/radial distribution function (RDF) [1,19,20], which is commonly used in statistical mechanics to describe the structure of dense and isotropic systems.

Selecting a suitable spatial scale s in Ripley’s K function remains a topic of discussion. While plotting K(s) or its derivative allows us to see at which spatial scales the clustering tendency compared with a uniform distribution is relatively large, picking a discrete set of spatial scales at which clustering explicitly takes place requires rigorous statistical analysis [21]. Regardless, the discrete nature of the neighbourhoods used in Ripley’s K function and the RDF is not well aligned with the way in which animals are considered to perceive their neighbourhood [22]. Generally, in for example attraction/repulsion models [23], the relative distance to the neighbours is far more important than whether the neighbours fall within some discrete distance bounds [24]. As such, a method that quantifies clustering scales with continuous instead of discrete distance-dependent weights to describe the effect of neighbours on a focal individual would be a more accurate representation of reality.

Recent studies have increasingly emphasized the need for more sensitive and spatially explicit methods to characterize aggregation. One such method is the *spatially explicit aggregation index* (SAI) [25], which accounts not only for group sizes but also for the spatial arrangement of individuals within groups. When detecting changes in aggregation for ungulates, the SAI has been shown to be more sensitive than the traditional *overcrowding index* [26], underscoring the ecological significance of internal group organization. However, the SAI provides only a single summary value for an entire dataset at a fixed aggregation scale, limiting its utility in settings where multiple scales of aggregation coexist.

A wide range of other summary statistics have been used to describe aggregation, including pairwise and nearest-neighbour distances. A comparative study evaluated the sensitivity of various aggregation metrics to behavioural changes in a population of elk [27]. Two of the most responsive metrics were based on *kernel density estimation* (KDE) [28,29], a method that also forms the foundation of our approach. These KDE-based metrics assess aggregation by measuring the area and total density within utilization polygons derived from different density levels of the estimated surface. Among the metrics tested, these simple KDE-derived measures proved particularly sensitive to behavioural variation, demonstrating that KDE can serve as a powerful tool for analysing spatial aggregation. However, because they reduce the KDE surface to a single threshold-based value, these metrics may overlook important spatial patterns arising from more complex or multi-scale forms of aggregation.

In this paper, we present a parameter-free density-based method to compute a summary characteristic that describes animal clustering at different spatial scales. Our approach bears similarities to Ripley’s K function and more specifically the RDF, but aims to address the following two drawbacks: (i) manually choosing the spatial scale and (ii) having discrete neighbourhoods. We use KDE [28,29] as an addendum to the RDF in an attempt to more accurately model that the way in which animals perceive their spatial neighbours is distance-dependent. KDE has been used for a variety of different applications beyond merely estimating a probability density function: from outlier detection [30] to discriminant analysis [31].

Our method produces a function that is dependent on a parameter representing the spatial scale of clustering in a similar way to the K function and the RDF. We show how to compute a discrete set of meaningful spatial scales from the topological features of this function, such that spatial scales need not be manually selected. Lastly, we test our methods on a number of artificial datasets that exhibit various different (hierarchical) clustering characteristics, as well as a real-world spatial dataset of African bush elephants (*Loxodonta africana*).

## 2. Material and methods

### 2.1. Metric calculation

Our approach to calculating clustering values is based on the density of the point set. In order to measure the density of a dataset, we use KDE [28,29]. KDE is classically used to reconstruct an estimate of the probability density function based on a finite sample. Given an input point set P in ${\mathbb{R}}^{2}$ and parameter σ, KDE uses *kernels* of width σ to construct a function ${\text{KDE}}_{P,\sigma }:{\mathbb{R}}^{2}\to {\mathbb{R}}^{+}$ such that ${\text{KDE}}_{P,\sigma }(x,y)$ estimates the density of P at position (x,y).

To obtain a KDE function, we must choose a kernel function ${\text{Ker}}_{\sigma }:{\mathbb{R}}^{2}\to {\mathbb{R}}^{+}$ that captures the influence of a single point on the density of the data. For our method to accurately describe animal clustering, the chosen kernel function needs to match the perception neighbourhood of each animal and thus be monotonically non-increasing and have circular level-sets at every height. Let σ denote the *kernel width* and ‖(x,y)‖ the Euclidean norm of (x,y). The parameter σ is used to determine the size of the area of influence of each point and is often referred to as the *kernel width*. Examples of common kernel functions are as follows [32]:

**Figure:**
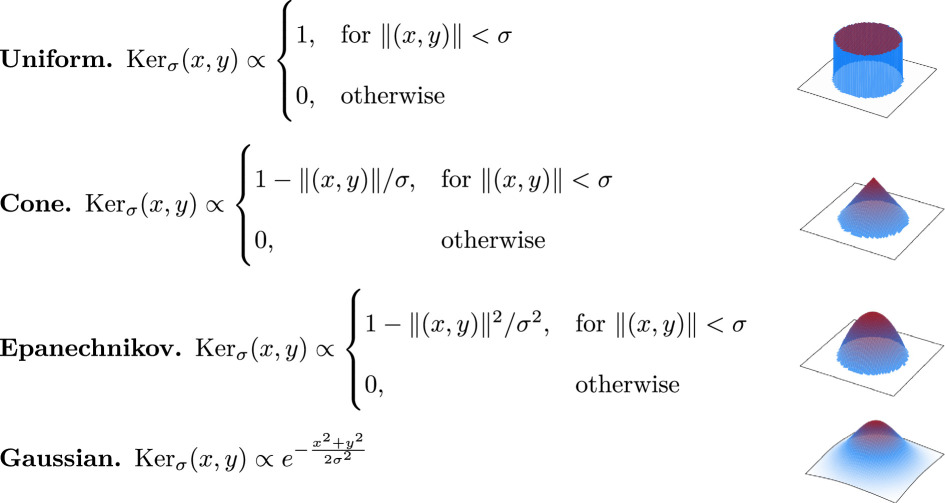


Then, we compute the KDE function as


$${\text{KDE}}_{P,\sigma }(x,y)=\frac{1}{n}\sum_{p\in P}{\text{Ker}}_{\sigma }(x-x(p),y-y(p))\;\text{for }(x,y)\in R^{2},$$

where x(p) and y(p) denote the x- and y-coordinates of point p, respectively. This results in a density function that has high values where points are dense and low values where points are sparse. Because the function generated in this way has two spatial input parameters and returns only positive values, it resembles a three-dimensional terrain. We often refer to the function ${\text{KDE}}_{P,\sigma }$ as the *density terrain* or *density surface*.

After obtaining the density surface, given density function ${\text{KDE}}_{P,\sigma }$ for point set P in a domain with area A and kernel width σ, we compute the clustering metric $M_{P}(\sigma )$ as


$$M_{P}(\sigma )=\frac{A}{n\cdot \sigma }\sum_{p\in P}{\text{KDE}}_{P\setminus \{p\},\sigma }(x(p),y(p)),$$

where P∖{p} is point set P after removal of point p.

We use kernel width σ as a descriptor of the spatial scale at which our metric measures clustering. As the metric sums the values of the density surface over all points, high values of $M_{P}(\sigma )$ should indicate that the point set is clustered at spatial scale σ. As such, calculating the clustering metric $M_{P}(\sigma )$ for a range of different kernel widths allows us to make comparisons on how clustered different point sets are at different spatial scales. Observe that this metric, when using the uniform kernel function, is $M_{P}(\sigma )=K(\sigma )/\sigma$, where K is Ripley’s *K* function.

Intuitively, the value of $M_{P}(\sigma )$ reflects the average local density of points, expressed as a fraction of the total number of points, within a distance σ from a randomly chosen point. Nearby points contribute more strongly according to the kernel function. To account for the fact that larger kernels naturally produce higher values, the result is normalized by dividing by σ. Because the value of $M_{P}(\sigma )$ depends on many different variables, it is best suited for comparative analysis across spatial scales rather than being interpretable in isolation.

While our approach uses a scalar kernel width parameter σ, we note that it is also possible to use a bandwidth matrix in multivariate KDE [33]. A bandwidth matrix allows the kernel to adapt to anisotropies or correlations in the data, providing more flexibility in modelling the local structure of point distributions. However, given that our underlying metric space is spatial and has two uncorrelated dimensions of the same scale, the scalar bandwidth has a clear geometric interpretation: it defines an isotropic perception range for each animal. Additionally, using a scalar parameter simplifies the analysis and reduces sensitivity to overfitting, which is especially relevant for ecological studies where the sample is often limited in size or unevenly distributed. Therefore, we use common symmetric univariate kernels parametrized by the distance to the kernel centre.

Clearly, the choice of a kernel function influences the exact output values of $M_{P}$. In this context, the choice of kernel function should represent the perception neighbourhood of each animal. Therefore, the kernel function should reflect that perception is symmetrically reduced as the distance to the centre increases, which makes the uniform kernel (as is also used in Ripley’s *K* function and the RDF) a discrete and very simplified representation of this decreasing perception field. The linear decrease of the cone kernel represents this decrease more smoothly, but this function does not describe that the decrease in the chance to perceive an animal accelerates over distance [34–37]. Therefore, the Gaussian kernel would fit the perception neighbourhood of most animal species better, but as this kernel does not have a finite base, it is not as trivial to relate the kernel width σ directly to spatial scales (as can be done with the other kernels) without truncating the kernel at an arbitrary distance (thereby effectively introducing another parameter). As such, for most of our analysis, we use the Epanechnikov kernel, which strikes a nice balance between computational simplicity and being representative of most animal species’ perception neighbourhood. However, we also perform our analysis once with each of the four different symmetric kernel types described above to quantitatively compare their accuracies in computing the spatial clustering scales.

#### 2.1.1. Finding meaningful spatial scales

Instead of manually selecting a value of σ, we want to automatically detect values for σ at which the point set is clustered. Typically, this is achieved by comparing the metric value with the expected metric value of completely randomly distributed input data. In our case, this complete spatial randomness (CSR) baseline is dependent on the chosen kernel function and can be computed as


$$\begin{array}{r} \text{CSR}(\sigma )=\frac{\int \int_{\;D}{\text{Ker}}_{\sigma }(x,y)dxdy}{\sigma }, \end{array}$$

where D is the domain in which ${\text{KDE}}_{P,\sigma }$ exhibits non-zero values. The dataset is then considered to be ‘clustered’ for values of σ where $M_{P}(\sigma )>\text{CSR}(\sigma )$. Therefore, rather than providing a discrete set of spatial scales at which the data are clustered, comparison with a CSR baseline yields a range of values at which the data are considered to be clustered.

To obtain a discrete set of clustering spatial scales, observe that increasing σ will generally result in lower values of $M_{P}(\sigma )$, because we divide by σ in the computation of $M_{P}(\sigma )$. The metric value can increase only when increasing σ results in a significant number of points entering each other’s neighbourhoods. As such, even though $M_{P}(\sigma )$ describes clustering behaviour *up to* scale σ, the value of $M_{P}(\sigma )$ will increase with σ approximately as long as σ is smaller than the cluster diameter and will stop increasing after it exceeds the cluster diameter. According to the above-mentioned argument, therefore, we can use our method to yield information about the diameter of isotropically shaped clusters. As such, intuitively, the local maxima of $M_{P}(\sigma )$ indicate the spatial scales at which maximal clustering takes place. Combining this observation with the CSR baseline, we select the local maxima of $M_{P}$ of which the value lies above the CSR baseline as the spatial scales at which a meaningful degree of clustering appears.

This method of detecting relevant spatial scales is not suitable for Ripley’s *K* function, as it monotonically increases with σ. Conversely, it is possible to apply this method to the RDF or functions that are proportional to it. However, because of its discrete nature, the RDF is very noisy by definition. Therefore, using topological features of the function to infer meaningful clustering scales yields many spurious spatial scales. To this end, we demonstrate the merit of our kernel smoothing approach over the RDF by comparing the results of using an RDF to detect relevant spatial scales with the results of our method when using the smooth Epanechnikov kernel.

### 2.2. Data collection

#### 2.2.1. Artificial data

We analyse how the performance of our method is dependent on a number of clustering characteristics using artificial datasets. These characteristics are

— the number of clusters c,
— the number of points per cluster m,
— the cluster diameter d,
— the number of spatial scales at which clustering occurs.

**Figure 1.**
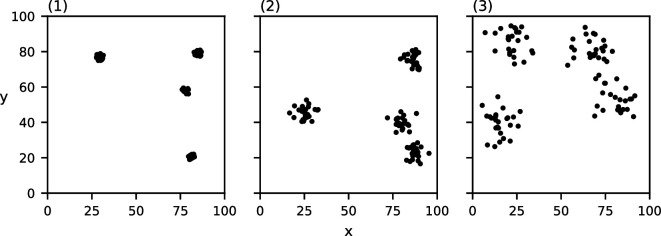
Three point sets consisting of c=4 clusters of m=25 points each with varying cluster diameters: (1) d=3, (2) d=10, (3) d=20.

**Figure 2.**
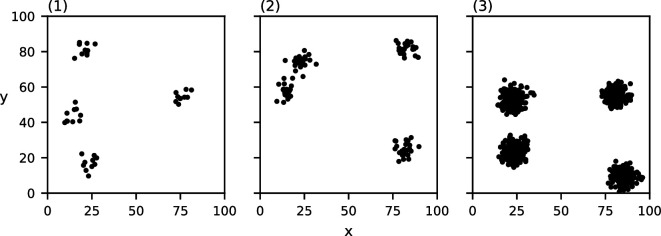
Three point sets consisting of c=4 clusters with a diameter d=10 each of a varying number of points: (1) m=10, (2) m=25, (3) m=250.

**Figure 3.**
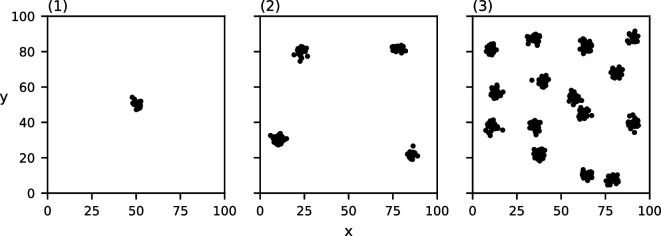
Three point sets consisting of clusters of m=25 points each with a diameter d=5 each with a varying number of clusters: (1) c=1, (2) c=4, (3) c=15.

**Figure 4.**
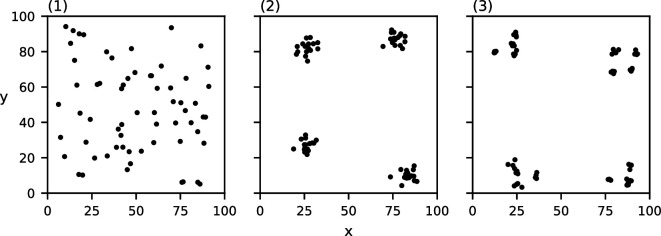
Three point sets of 64 points each clustered at a different number of spatial scales: (1) no clustering, (2) one level of clustering, (3) two levels of clustering.

For each of these characteristics, we construct three datasets in which the chosen characteristic varies. To construct the clusters in each of these datasets, we place c cluster centres in a regular grid and then perturb their location uniformly randomly while maintaining pairwise spatial separation. Next, for each cluster centre, we place m points according to a normal distribution centred at the cluster centre with s.d. d/6. We pick this s.d. to ensure that approximately 95% of the points lie within the chosen cluster diameter. The generated point sets can be found in figures 1–4. Note that the data in figure 4(2) are clustered at one scale with c=4, m=16 and d=10, and the data in figure 4(3) are clustered at two scales with c=16, m=4 and d=4, where the clusters of diameter d=4 are recursively clustered in larger clusters of diameter d=20. The data points in figure 4(1) are placed according to a uniform distribution.

#### 2.2.2. Real-world data

**Figure 5.**
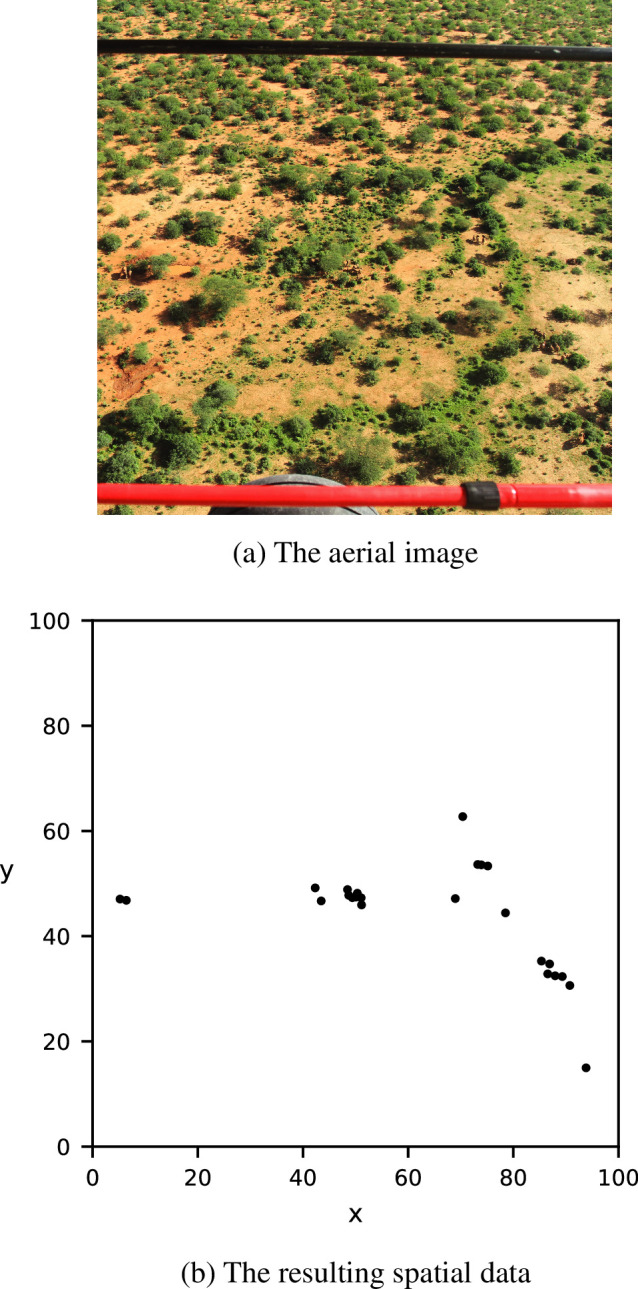
The real-world dataset was obtained by manually annotating an aerial image of African bush elephants in Kenya [38].

We additionally quantitatively compare our method using the Epanechnikov kernel with Ripley’s *K* function and the RDF using real-world data collected in March 2014 in the Tsavo National Parks, Kenya. We use a subset of the original dataset, consisting of location data of 24 elephants (*L. africana*) obtained from an aerial image that were manually taken by human observers upon spotting the animals. The aerial image was manually processed into spatial data by placing a point on the approximate centre point of each animal in the image. The original aerial image, as well as the resulting dataset, can be found in figure 5. For more details on the data collection process, see [38].

## 3. Results

### 3.1. Artificial dataset

#### 3.1.1. Kernel types

**Figure 6.**
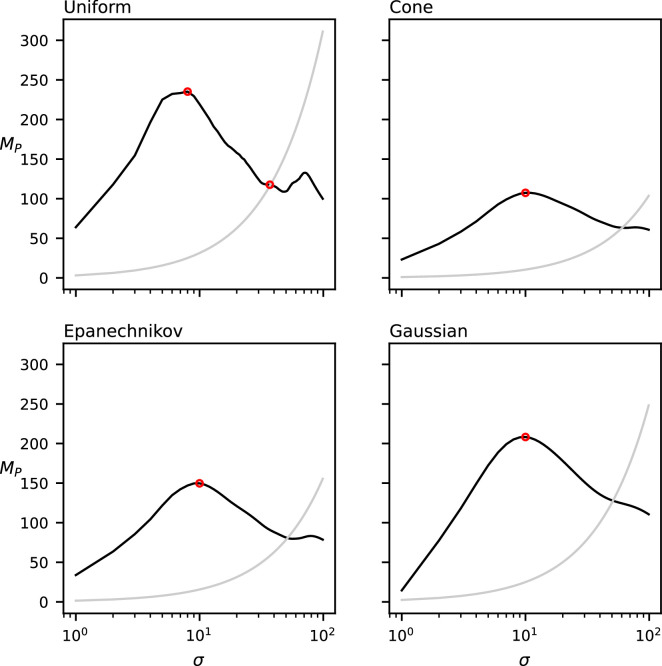
$M_{P}(\sigma )$ for the four different kernel functions of the dataset in figure 1(2). The grey curve indicates the CSR baseline. Local maxima above CSR are annotated in red.

To evaluate the effect of the kernel function on $M_{P}$, we compute $M_{P}$ over the dataset in figure 1(2) for each of the four different kernel functions shown in the previous section: the uniform, cone, Epanechnikov and Gaussian kernels. These results can be found in figure 6.

The four functions produced by each of these kernel functions are relatively similar. The uniform and Gaussian kernels produce generally higher function values than the cone and Epanechnikov kernels. Additionally, the function computed by the uniform kernel is noisier than those of the other three kernel types. However, all four of the tested kernel types seem to produce similar results for the chosen dataset.

For each of the four chosen kernel types (except the uniform kernel), our method detects only a single relevant spatial scale (recall that we deem a spatial scale relevant if it induces a local maximum in $M_{P}$ of which the value is higher than the CSR baseline) that is approximately equal to the chosen diameter of the clusters in the data (c=10); the uniform kernel finds two relevant spatial scales ($\sigma =8,M_{P}(8)=235.3$; $\sigma =37,M_{P}(37)=117.7$), the prior of which appears at a slightly lower scale than those in the cone kernel ($\sigma =10,M_{P}(11)=107.4$), the Epanechnikov kernel ($\sigma =10,M_{P}(10)=149.8$) and the Gaussian kernel ($\sigma =10,M_{P}(10)=208.2$).

#### 3.1.2. Clustering characteristics

The following results are obtained using the Epanechnikov kernel.

**Figure 7.**
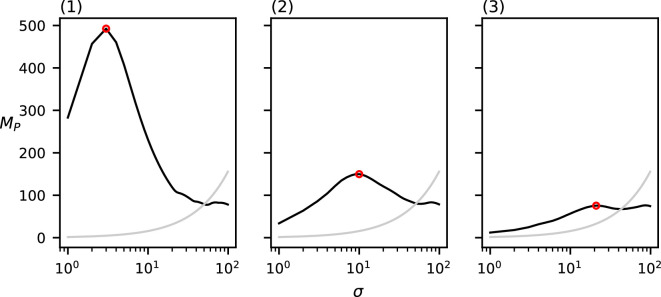
$M_{P}(\sigma )$ of the three datasets from figure 1 with varying cluster diameters ((1) d=3, (2) d=10 and (3) d=20)). The grey curve indicates the CSR baseline. Local maxima above CSR are annotated in red.

The values of $M_{P}$ for the three point sets from figure 1 with varying cluster diameters can be found in figure 7. Each of the three datasets induce a single relevant spatial scale at (1) $\sigma =3,M_{P}(3)=492.5$*,* (2) $\sigma =10,M_{P}(10)=149.8$ and (3) $\sigma =21,M_{P}(21)=75.52$. Note that these detected spatial scales are (nearly) equal to the chosen cluster diameter in the corresponding dataset. Of additional note is the clustering value for each of these three relevant spatial scales; the value of $M_{P}$ at the detected relevant spatial scales seems to decrease as the cluster diameter increases.

**Figure 8.**
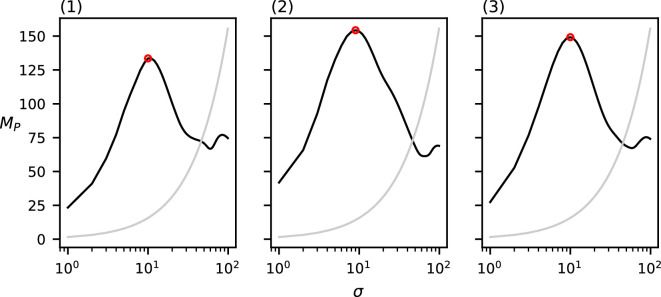
$M_{P}(\sigma )$ of the three datasets from figure 1 with varying numbers of points per cluster ((1) m=10, (2) m=25 and (3) m=250)). The grey curve indicates the CSR baseline. Local maxima above CSR are annotated in red.

The values of $M_{P}$ for the three point sets from figure 2 with varying numbers of points per cluster can be found in figure 8. Again, each of these three datasets induce a single relevant spatial scale at (1) $\sigma =10,M_{P}(10)=133.5$*,* (2) $\sigma =9,M_{P}(9)=154.3$ and (3) $\sigma =10,M_{P}(10)=149.2$. Thus, the detected spatial scale in each of these three datasets is (nearly) equal to the chosen cluster diameter of 10. Notably, all three functions are similar, and those of (2) and (3) are nearly identical.

**Figure 9.**
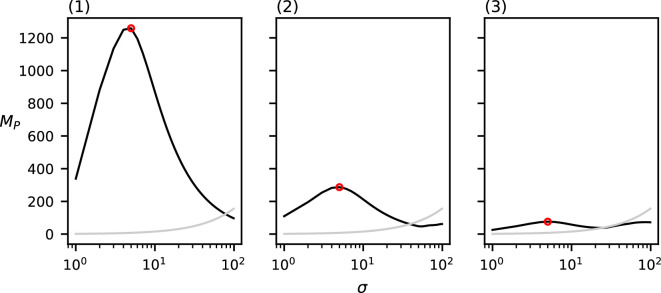
$M_{P}(\sigma )$ of the three datasets from figure 2 with varying numbers of clusters ((1) c=1, (2) c=4 and (3) c=15)). The grey curve indicates the CSR baseline. Local maxima above CSR are annotated in red.

The values of $M_{P}$ for the three point sets from figure 3 with varying numbers of clusters can be found in figure 9. Each of these three datasets induce a single relevant spatial scale at σ=5, with respective values (1) $M_{P}(5)=1258.9.3$, (2) $M_{P}(5)=286.9$ and (3) $M_{P}(5)=75.9$. The detected spatial scale in each of these three datasets is exactly equal to the chosen cluster diameter. Note that the value of $M_{P}$ at this detected spatial scale is reduced as more clusters are placed.

**Figure 10.**
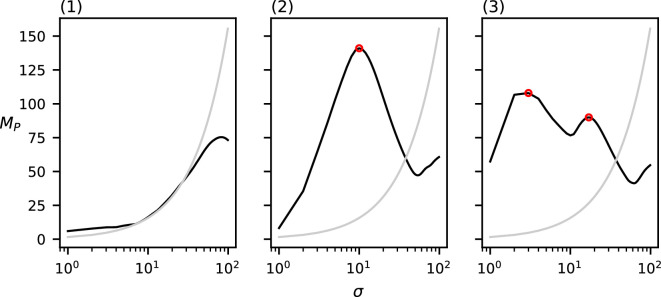
$M_{P}(\sigma )$ of the three datasets from figure 4 with varying clustering levels ((1) no clustering, (2) one level and (3) two levels)). The grey curve indicates the CSR baseline. Local maxima above CSR are annotated in red.

Lastly, the values of $M_{P}$ for the three point sets from figure 4 with varying numbers of clustering scales can be found in figure 10. Our method correctly detects no relevant spatial scales in (1) and a single relevant spatial scale in (2) $\sigma =10,M_{P}(10)=141.0$*,* which is equal to the chosen cluster diameter in (2). In (3), our method detects two relevant spatial scales: one at $\sigma =3,M_{P}(4)=108.0$, which approximates the diameter of the small sub-clusters, and one at $\sigma =17,M_{P}(18)=90.0$*,* which approximates the diameter of the larger clusters.

**Figure 11.**
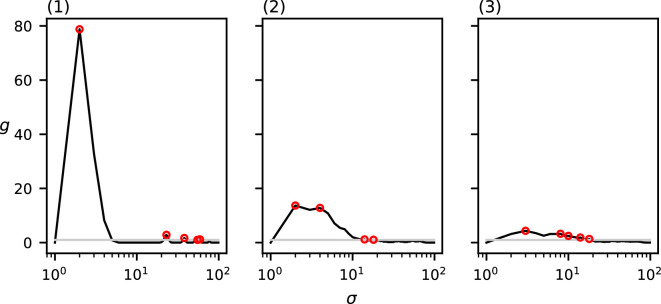
The RDF of the three datasets from figure 1.

**Figure 12.**
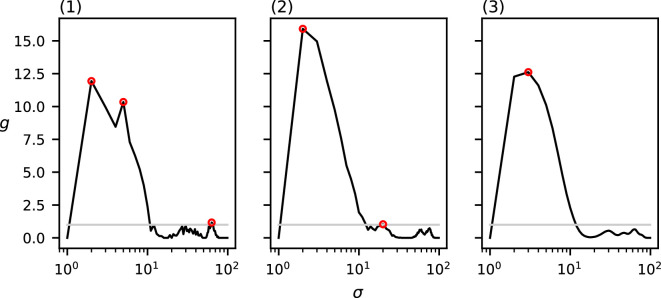
The RDF of the three datasets from figure 2.

**Figure 13.**
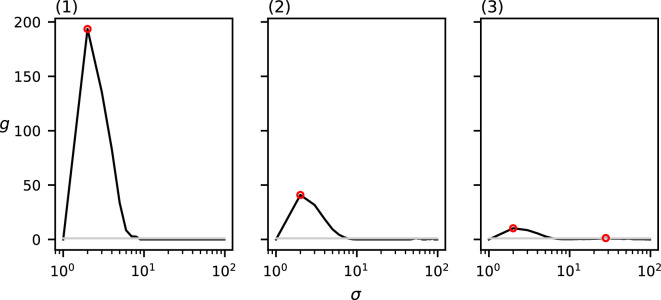
The RDF of the three datasets from figure 3.

**Figure 14.**
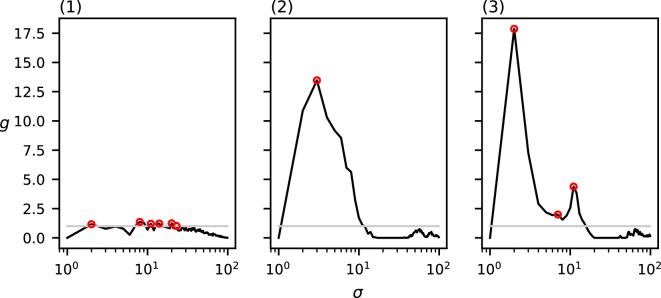
The RDF of the three datasets from figure 4.

Observe that in all of the above-mentioned results, the relevant spatial scales detected by our method predict the input cluster diameter with high accuracy. In figures 11–14, we show that the detected spatial scales, when performing the same analysis as in figures 7–10 with the RDF, exhibit a lower accuracy and often detect spurious spatial scales.

### 3.2. Real-world data

**Figure 15.**
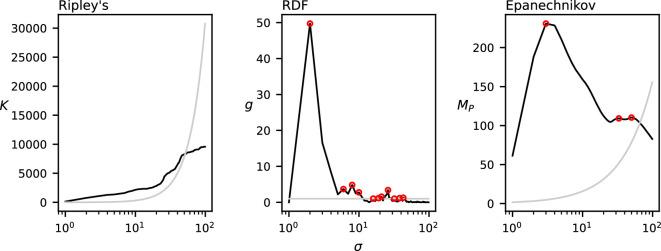
The output on the real-world dataset of (1) Ripley’s *K* function, (2) RDF g(σ) and (3) $M_{P}(\sigma )$ using the Epanechnikov kernel. The grey curve indicates the CSR baseline. Local maxima above CSR are annotated in red.

Figure 15 shows the values of Ripley’s *K* function, the RDF and $M_{P}$ with the uniform Epanechnikov kernel on the dataset in figure 5. Ripley’s *K* function in (1), when compared with the CSR baseline, shows for which range of spatial scales the data are considered to be more clustered than random data. However, we cannot use our method of detecting a discrete set of relevant spatial scales on this function. [Panels are not numbered in figure 15] shows the RDF. There are a large number of local maxima in this function above the CSR baseline, which is not representative for the clustering behaviour in the data, and can probably be attributed to noise. The application of the smoother Epanechnikov kernel results in the function in [Panels are not numbered in figure 15], which reduces noise in the function and results in only three relevant spatial scales at σ=3,33,50*,* which fits the trends of the function and the cluster diameters of figure 5b better.

## 4. Discussion

In this paper, we demonstrate how to quantify animal clustering at different spatial scales using a parameter-free method. Our summary characteristic successfully computes the relevant spatial scales of clustering that were used to produce artificial spatial point patterns. Our approach bears some similarity with the popular Ripley’s *K* function [17] and the RDF [1,20], which, similar to our approach, uses the average proportion of points in a fixed neighbourhood of any one entity. However, contrary to the *K* function and the RDF, we use kernels to assign weights to the points in these neighbourhoods, based on the distance to the neighbourhood centre. This more closely resembles how animals are considered to perceive their neighbourhoods [22]. But our approach can be applied more generally as well: changing the kernel function used to calculate $M_{P}(\sigma )$ allows the function to be tailored specifically to the desired application area.

Analysis of an artificial dataset shows that the metric functions for four different kernel functions look visually quite similar. The main difference lies between using a smooth kernel and using a discrete neighbourhood in the uniform kernel. The remainder of the analysis was performed using the Epanechnikov kernel. The reason for choosing the Epanechnikov kernel is that it has a finite base, which means that using σ (the radius of this finite base) to describe the spatial scale is more accurate with the Epanechnikov kernel than when truncating a Gaussian kernel at an arbitrary distance. Generally speaking, though, the specific choice of ‘smooth’ monotonically decreasing kernel did not impact the accuracy of detecting the spatial clustering scales very much. However, all three ‘smooth’ kernel types were far more accurate than using discrete/uniform neighbourhoods, which demonstrates the quantitative improvement that our density surfaces approach offers compared with a continuous implementation of Ripley’s *K* function or the RDF.

The remaining four analyses on artificial data investigate the results of four different clustering characteristics on the metric function and on the detected relevant spatial scales: (i) cluster diameter, (ii) number of points per cluster, (iii) number of clusters, and (iv) number of clustering levels. These results all demonstrate the general applicability of our method for point patterns with these varying characteristics.

Firstly, when varying the cluster diameter, figure 7 shows that the detected relevant spatial scale almost exactly corresponds to the chosen cluster diameter. This makes sense, as growing kernels of this scale centred at individuals at the edge of the clusters no longer introduce new individuals from that same cluster into the kernel. The metric value at the detected spatial scale decreases with the cluster diameter. This is also as expected, as the intra-cluster density is lower, and therefore the kernel value of each point within the same cluster is also lower for all points.

Secondly, figure 8 shows that the number of points per cluster seems to have little effect on the resulting metric function. Because, in the metric calculation, we divide by the number of points, this is according to expectation. For the dataset with only 10 points per cluster, we can clearly see how, even for very low numbers of points per cluster, our method is able to closely estimate the cluster diameter.

Thirdly, figure 9 shows that the number of clusters in the data seems not to have an effect on the detected relevant spatial scales. For all three of the datasets with varying numbers of clusters, the detected spatial scale corresponds exactly with the chosen cluster diameter. The metric value at this detected scale, however, is negatively correlated with the number of clusters in the data. This is to be expected, as the fraction of points in each cluster is lower for higher numbers of clusters.

Lastly, figure 10 shows that our method is also able to detect multiple clustering spatial scales in a single dataset and correctly finds no clustering spatial scales in a uniformly distributed dataset. Note that, for all of the above-mentioned experiments, the area outside of the domain is considered to be empty. As such, most of the metric functions exhibit another local maximum at the domain size, when the entire dataset is considered to be one big cluster. Comparing the metric value of this local maximum with the CSR baseline ensures that this local maximum is not detected as a relevant spatial scale, because only function maxima above the CSR baseline are considered relevant clustering spatial scales.

For the real-world dataset, figure 15 shows that Ripley’s *K* function, the RDF and our method with the Epanechnikov kernel consider the same range of spatial scales to be more clustered than the CSR baseline. However, Ripley’s *K* function does not provide a discrete set of relevant spatial scales at which the animals are clustered. The local maxima of the RDF could intuitively correspond to the relevant clustering spatial scales. However, because of the discrete nature of the neighbourhood considered in the RDF, this function is noisy and exhibits many local maxima closely together. Using our proposed smoother Epanechnikov kernel reduces the number of detected spatial scales from seven to three at σ=3,33,50, which matches better with the clustering patterns when interpreting figure 5 optically.

For future research, it is possible to build upon our proposed method by compensating for the domain edge through edge correction. For Ripley’s *K* function and the RDF, there is extensive literature describing how to perform edge correction [39,40], usually by weighting each point by the fraction of the area of its radius-σ kernel that falls outside of the domain. This could be useful when these methods are used for interpreting structure in dense isotropic point patterns in, for example, particles in gases or liquids, as these structures are often repeating beyond the boundaries of the domain in which the function is constructed.

Theoretically, it is possible to perform similar edge corrections for different kernel functions by weighting each point by the amount of volume under each individual kernel function that falls outside of the domain, rather than the area of the kernel base. In our application of clustered animals, however, there is often a lower degree of structure in the point patterns. More importantly, the boundaries of our domain enclose the group of animals we want to study, and the areas outside our domain are assumed to be empty of relevant animals. This not only removes the necessity for edge corrections but also makes it undesirable.

Besides this consideration, there are two other practical considerations to take into account when applying our method to compute clustering at different scales. Firstly, to cover all potentially relevant clustering scale levels, it is sufficient to consider the range of kernel widths used for computing $M_{P}(\sigma )$ profile upper bounded by the domain size and lower bounded below the smallest inter-individual distance of the point set. Secondly, kernels should have a circular shape in the (x,y) plane to make sure the $M_{P}(\sigma )$ values are independent of the (x,y) orientation of the domain. Consequently, our method assumes that animal groups are isotropically shaped. However, for most kernels, moderate deviations from isotropic are allowed, as they exhibit small values at the kernel boundaries.

While our method provides a robust and flexible way to quantify clustering and detect relevant spatial scales, there are scenarios in which additional refinements may be necessary. For instance, in datasets where multiple individual groups exhibit distinct clustering scales, e.g. in response to different local conditions, the global metric function may blend these patterns into a single curve, potentially obscuring meaningful differences. Similarly, the current approach assumes a single kernel function is appropriate for all points, which may not hold in cases where individual animals perceive their surroundings differently, e.g. when studying multiple species simultaneously that have varying sensory capabilities or local environments. Incorporating adaptive or locally defined kernels, or developing multi-scale decompositions of the metric function, could offer richer insights in such settings. Another promising direction is the integration of temporal dynamics, allowing clustering patterns to be tracked and quantified over time as animal distributions evolve. If the input data include a temporal component, it also becomes possible to incorporate directional information, like the animals’ heading, into the analysis. This would enable more flexible modelling of perception neighbourhoods, which may not be symmetric but instead biased in the direction of movement. Capturing such directional sensitivity would require the use of anisotropic kernel shapes. While this added flexibility could yield more accurate representations of individual perception, it would also introduce additional complexity into the analysis and interpretation of the results.

Identifying relevant clustering scales enables concrete ecological interpretations. In the elephant case study, the smallest detected scale probably corresponds to tight-knit social units within the herd, while larger scales may reflect social aggregations or shared habitat use at the herd or population level. This information could help assess social structure, resource sharing or responses to environmental pressures. More generally, identifying multiple clustering scales can inform management strategies for species that exhibit hierarchical spatial organization, such as elk [41] or sage-grouse [42]. Our techniques can also be used in other application areas in which entities exhibit hierarchical clustering, such as the distribution of termite mounds [43] or alloy structures in material sciences [44]. The parameter-free nature of the method makes it especially well-suited for applications where relevant scales are not known *a priori* or where systems are expected to exhibit complex, nested spatial structures.

Until now, no method has been available to quantify the degree of clustering of animals in a parameter-free way and precisely derive the spatial scales at which these animals are clustered. Our method makes it possible to, for example, compare the specific spatial clustering scale(s) between different groups of animals. Moreover, our approach is able to more accurately detect these scales than a continuous implementation of Ripley’s *K* function or the RDF is able to do, by using monotonically decreasing kernels that better describe the perceptive field of animals. With this result, our paper can contribute to a better understanding of the stimuli underlying changes in the spatial patterns of animals.

## Data Availability

All scripts and data used for these analyses are uploaded to the 4TU.ResearchData repository at [45].
